# How the ASCENT model can help optimize exposure therapies for Autistic clients

**DOI:** 10.3389/fpsyt.2025.1569882

**Published:** 2025-11-05

**Authors:** Amara Brook

**Affiliations:** ^1^ University of Nevada, Reno, Reno, NV, United States; ^2^ Flow Psychological Services, Reno, NV, United States

**Keywords:** exposure, anxiety, autistic, autism, ASCENT, neurodiversity, OCD, social anxiety

## Abstract

Exposure therapies are very effective for alleviating anxiety, yet limited research has focused on optimizing their effectiveness for Autistic clients. This “Perspective” article describes how the ASCENT model can guide tailoring exposure therapies to be more effective and affirming for Autistic clients. This model proposes supporting clients in Autonomous and Affirming goal setting (A), adapting for differences in Sensory processing, Stimming, Structure, Special interests (S), Communication (C), and Executive functioning (E), practicing with Neurohumility (N), and being Trauma informed (T). Because the ASCENT model is based on traits of Autistic clients and effective ways of working with them, it can help guide tailoring a wide variety of anxiety treatments. This article provides examples of how the ASCENT model can be applied to increase the effectiveness of several different types of exposure therapies, as well as articulating when exposure is not appropriate and accommodation is necessary instead.

## Introduction

Anxiety can save our lives and prevent other harm by motivating us to avoid danger (1, 2). However, when anxiety is too high, it causes avoidance of things that are not dangerous, robbing us of valuable experiences (3), and ironically, maintaining the anxiety (4, 5). Exposure therapy can help reduce fear when fear is out of proportion to actual danger. It involves intentionally experiencing feared stimuli until fear decreases because no harm occurs. Exposure may reduce reactivity to feared stimuli through habituation (6), reduced response with repeated exposures to a stimulus (7, 8), and inhibitory learning, building a safe association with a stimulus that inhibits the fear association (9). Intentionally exposing ourselves to things we are scared of that are not dangerous is an effective way to learn that they will not hurt us (4, 9). Despite extensive research on the effectiveness of exposure therapies for alleviating anxiety (10–15), high levels of anxiety in Autistic people (16–18), negative consequences of anxiety for Autistic people (19, 20), and differences between Autistic and non-Autistic people (21, 22), less research has focused on how to optimize the effectiveness of exposure therapies for Autistic clients (23–26). There is some evidence that exposure therapies can help some anxious Autistic clients some of the time (25). Yet without tailoring to the needs of Autistic clients, these therapies could perpetuate ableism, prejudice and discrimination against disabled people (27), and be ineffective or even harmful (28). What do we need to consider in tailoring exposure therapies for Autistic clients?

## The ASCENT model

My colleagues and I developed The ASCENT model to guide tailoring Dialectical Behavior Therapy (DBT) to be more effective and affirming for Autistic clients (29–31). This model is based on honoring and accommodating differences in Autistic individuals. Preliminary research findings support these recommendations for tailoring DBT (32, 33). McVey and colleagues (32) used the Discover Design Build Test (DDBT) framework (34) to identify needed modifications to DBT from Autistic young adult participants. Some adaptations, which align with the ASCENT model described below, include incorporating an anti-ableist lens, adjusting for Autistic characteristics such as sensory differences, special interests, need for structure, communication differences, and executive functioning, and educating DBT clinicians about Autistic differences and invalidation history. While these initial results are promising, further empirical research is needed. Additional research supporting the model is cited below.

### A — Autonomous and affirming goal-setting

To be affirming and not ableist, therapy must be guided by the client’s goals (35) rather than neurotypical-normative, ableist goals that aim to make the client appear less Autistic for the comfort of others. Both therapists and clients have often received and internalized ableist “shoulds” from others (27) (e.g. “I/she shouldn’t be so afraid of loud sounds.” Therapists and clients can curiously, collaboratively question ableist assumptions. An appropriate target for exposure is a fear out of proportion to actual danger that the client wants and is able to change, not an Autistic need that others think the client should change.

### S — Sensory, stimming, structure, SPINs

Sensory: Autistic clients often have differences in sensory processing, including being more sensitive (hypersensitivity) or less sensitive (hyposensitivity) to sensory input than others (21, 22). Furthermore, Autistic clients do not habituate to sensory stimuli as readily as others do (28). Instead, exposure to sensory stimuli continues to hurt. Thus, therapists need to accommodate Autistic clients’ sensory hypersensitivities rather than targeting them using exposure (36). Additionally, many Autistic clients experience hyposensitivity to interoceptive signals, sensory signals from inside the body such as heartrate, temperature, thirst, hunger, fatigue, or needing to use the restroom (37, 38), along with alexithymia, difficulty identifying and describing emotions (39–42). They may need support with emotion recognition (43–48), including different ways of rating level of emotions.

Example: Specific phobias are common in Autistic people (49), and exposure therapy is the most effective treatment for phobias (50–52). It is critical to discern whether an Autistic client is hypersensitive to a stimulus, or has average sensitivity but is phobic of the stimulus (53). For example, if an Autistic individual seems afraid of and avoids mushrooms, is it because of an aversion to the texture, a sensory hypersensitivity? Or fear of dying if the mushroom is the wrong type, a phobia? Exposure is only appropriate if the primary problem is a phobia, not a sensory hypersensitivity. Sometimes, Autistic clients may develop phobias of things they associate with overwhelming sensory input, such as fear of gyms due to an association with loud noises. In this case, any exposures to the non-sensory stimulus (e.g. gyms) would need to be coupled with protection (e.g. ear protectors) from the sensory stimulus (e.g. loud noises).

Stimming: Autistic clients often seek sensory input, or “stimming”, to regulate and express themselves. “Stims” might be stereotypical, like rocking, hand-flapping, finger flicking, or immediate echolalia, or less obvious, like hair twirling, skin picking, finger tapping, reciting lyrics of songs or quotes from movies, or reading or watching the same media repeatedly (22, 54, 55). Because stimming serves important functions, accommodate stimming in the context of other exposures without targeting it as a “safety behavior.” If a stim is harmful, such as cutting, headbanging, or excessive skin picking, therapists and clients can curiously explore the functions the stim serves and replace it with a benign alternative stim that serves the same functions.

Structure: Autistic clients often need structure and routine (21, 22, 56), which it is important to honor (57). Collaborate with the Autistic client by being flexible and providing needed structure, routine, and predictability during exposures (43, 58).

SPINs: Autistic clients often have intense and/or unusual interests, known as “special interests” or “SPINs”, that are part of their identities and provide joy and motivation. Incorporating SPINs during exposure therapy can help build rapport and increase motivation (31, 58). Multiple studies found that CBT incorporating SPINs reduced Autistic children’s anxiety more than comparable CBT that did not incorporate SPINs (59, 60). Do not coercively use SPINs, such as withholding a SPIN until the client complies, because this could re-traumatize Autistic clients who have been socially manipulated by others (31).

Example: Autistic people are more likely to have OCD (61). Exposure and Response Prevention (ERP), in which clients encounter obsessions without engaging in compulsions, is a highly effective exposure-based treatment for OCD (62, 63). Many suggested adaptations of ERP for Autistic clients align with the ASCENT model (45–47, 61, 64). Distinguish between OCD obsessions versus Autistic SPINs, and between OCD compulsions versus Autistic Stimming (61). Generally, OCD obsessions and compulsions are experienced as unpleasant and ego-dystonic, whereas Autistic SPINs and stimming are experienced as pleasurable and ego-syntonic, though Autistic clients may have developed shame about them due to negative reactions from others. Also, distinguish between OCD obsessions versus justified dread of overwhelming sensory stimuli. Use ERP to target OCD obsessions and compulsions while accommodating Autistic SPINs, stimming, need for structure, and sensory sensitivities (45–47, 60).

### C — Communication

Autistic clients may have communication differences that require accommodation (22, 58, 65–73). Use direct, clear, literal language (45, 46, 74), though metaphors related to SPINs may be helpful (31, 60, 75). Allow more time to process questions before expecting a response and to answer detailed questions (45, 46, 60, 70, 76–83). Provide information visually and support non-spoken means of communication such as writing or drawing (25, 43, 47, 70). Accommodate aphantasia, difficulty visualizing, with tools such as virtual reality, which has reduced specific phobias in Autistic clients (84, 85).

### E — Executive functioning

Autistic clients have differences in executive functioning (86–88), and many also meet criteria for ADHD (89). Many exposure therapies have high executive functioning demands, including focusing on difficult material during sessions, independently completing and documenting exposures between sessions, and attending sessions consistently, on time. Autistic clients may need executive functioning supports (25, 43, 45) such as reducing distractions during sessions, longer sessions to reduce transition demands (90, 91), help planning exposures and using calendars and lists, providing important information in writing to support memory, and providing reminders about appointments. Greater parent involvement may help support executive functioning (25) if parents’ executive functioning needs (92, 93), are supported.

### N — Neurohumility

Autistic clients have neurodevelopmental differences and life experiences that are different from those of many therapists, and many have experienced unintentional invalidation by therapists. Neurohumility is an extension of cultural humility (94–99) that asks therapists to be aware that there is much they do not know and to be open to learning, radically humble, and validating to Autistic clients to repair and prevent further invalidation trauma (29–31, 100). Like cultural humility (96, 101), neurohumility is critical to developing a collaborative, trusting, effective therapeutic relationship with Autistic clients, which is the strongest predictor of positive outcomes in therapy (102, 103), and likely also reduces therapist microaggressions (104), helps the therapeutic relationship recover from cross-neurotype mistakes (98), and predicts better outcomes (105–107). Empirical research on neurohumility is in its infancy. A recent paper argued that humility is an essential component of neurodiversity-affirming therapy (108), a recent ethnographic study described the effectiveness of using a cultural humility approach for working with Autistic youth (109), and my colleagues and I have described the importance of neurohumility in our own lived experiences and clinical practices (29–31, 100).

### T — Trauma informed

Due to living in a culture not designed for their neurotype, most Autistic clients have experienced an extensive history of chronic traumatic invalidation (46, 110, 111), which causes hypervigilance for further invalidation and interferes with the ability to trust others including therapists who suggest exposures. Therapists working with Autistic clients need to be trauma-informed and highly validating in order to rebuild trust (31, 112). Only when the client trusts the therapist can the client make themselves vulnerable through exposure therapy.

### When exposure is contraindicated and accommodation is more appropriate

Appropriate exposure targets are things that are not harmful, that the client wants to be able to experience, and on which exposure will work to reduce fear. Exposure is contraindicated when it would be invalidating and/or ineffective. Because Autistic clients do not habituate to sensory stimuli as others do (28), using exposure to target sensory sensitivities is contraindicated because it would be both invalidating (denying sensory experiences and needs) and ineffective (inflicting more sensory pain rather than reducing fear) (25, 31). Similarly, exposures to communication or social situations in which clients are likely to be further traumatized, such as those that require picking up on subtle signals that they tend to miss, that do not provide them enough time to think before responding, that are too unstructured, or that involve others who are likely to exclude or reject them, are also invalidating and ineffective. When exposure is contraindicated, accommodate instead (25). Research aligned with the neurodiversity paradigm (113) has emphasized the need to create more accommodating social and physical spaces that align with the needs of Autistic people, rather than pushing them to suppress their needs to accommodate others, to their own detriment (114–116).

## Optimizing exposure with ASCENT model: case example

Robin is a 20-year-old Autistic college student. She seeks therapy at her college counseling center after her roommates expressed concern that she rarely leaves her room except to go to class and eat. In her intake questionnaires, she reports worries about many social situations. She wants to have friends but social mixers, such as the sorority rush that her mother and former therapist suggested, and other unstructured interactions with peers, have rarely gone well. She doesn’t know what to do, small talk is hard for her, and even if she does connect initially, the relationships seem to fade over time. Except for one close friend she had in K-12 school who was also neurodivergent, peers have laughed at and excluded her when she talks about her passionate interest in lizards and when she repeatedly uses lines from her favorite anime in conversation. The only time that peers accept her is when she hides who she is, which is exhausting, unsustainable, and leads to more shame. She has always connected better with older adults who appreciate her uniqueness. She also feels overwhelmed in noisy spaces, and more comfortable by herself in her room or in structured situations such as lectures and labs. She was diagnosed Autistic in high school and thought things would be easier for her in college, but they have not. Now she is fearful of and avoids most social interactions. How can her therapist help her most effectively?

Exposure for social anxiety involves voluntarily experiencing feared social situations, and may be effective when social fears are exaggerated (14, 117, 118). Social anxiety is much more prevalent among Autistic (up to 50%), than non-Autistic (7-13%) people (16, 119–121), and often more accurately reflects Autistic social reality, given experiences of social exclusion and rejection (120, 122–124). Therefore, exposure for social anxiety also needs to be tailored for Autistic clients to provide positive social experiences and avoid further social trauma.

Autonomous and Affirming Goal-setting – Center the client’s social goals rather than assuming neurotypical social goals (35). Unlike the stereotype of Autistic people as socially unmotivated loners, most Autistic people, like Robin, do want some social interaction and relationships (125–130). But they may prefer for their relationships to look different from a neurotypical norm in terms of number, frequency, format, and other aspects (131–135). Work collaboratively with Autistic clients to clarify and validate their social goals. Robin’s therapist, Steve, recognizes that Robin said she does want relationships and enjoyed a long-standing relationship with a peer in the past. So, he starts by asking her what she enjoyed about that relationship, including what they enjoyed talking about and doing together. They discuss and Steve validates what she wants socially, one or two close friends with whom she can focus on her interests in lizards and anime and be herself.

Sensory, Stimming, Structure, SPINs – Accommodate sensory needs, stimming, structure, and SPINs during social exposures with Autistic clients. Some social settings such as bars, restaurants, or large parties provide too much sensory stimuli for many Autistic clients (25, 74, 136), and they need either settings with less sensory stimuli, or to use tools such as ear filters. Social exposures such as group workouts, singing events, dancing events, or rollercoaster parks can accommodate stimming needs (74). And social exposures that accommodate need for structure (136) such as gaming (137, 138) or studying literature (139) may be more effective than unstructured ones such as mixers. Social exposures based on a SPIN (e.g. game nights, dancing events, group workouts, conventions, interest-based club meetings, lectures) are likely to be more enjoyable and likely to build connections. Steve validates Robin’s sensitivities to noise and they agree to choose social exposures that are quieter, and discuss ways to meet her sensory needs in louder situations (e.g. ear protectors). Steve notices that Robin often twirls her hair, taps her fingers, draws anime characters, and repeats lines from anime during their sessions, especially when their conversations veer into less comfortable territory. He curiously, nonjudgmentally asks her if these help her regulate, and she shares that they do. He also validates her interests in anime and lizards and need for structure in social situations. They agree to choose social exposures that accommodate these needs, including taking a herpetology class, and joining a lizard research lab and the Anime club.

Communication – Accommodate Autistic clients’ communication needs during social exposures. This could include interaction format, such as written rather than oral communication (140). Neurotype matching might also be helpful. Autistic people connect more easily with others who share their neurotype (67, 72, 74, 130, 141–144), so social exposures involving interactions with other Autistic people may be more positive and likely to foster close social connections. While there are times when Autistic clients need to interact with people of other neurotypes, social fears are more likely to be accurate in these interactions (120, 145) and they involve more minority stress (110) and exhausting camouflaging (74, 146, 147), which is related to increased depression and suicidality (148, 149). Helping socially anxious Autistic clients find supportive social environments that match their communication needs and where they unmask safely is essential (150). Recognizing that Autistic students often connect more easily with each other, Steve suggests the clinic’s Neuro-Connect group, which meets weekly and also happens to have other students interested in anime. It also supports different communication formats in addition to talking, such as writing, drawing, or sharing memes, depending on participants’ needs.

Executive Functioning – Autistic clients may need executive functioning support to plan, organize, and engage in social exposures. Steve and Robin collaboratively prioritize social exposures and discuss step-by-step how to approach each exposure. He invites her to share any concerns she has about them, including reaching out to him for help later if she encounters difficulties she didn’t anticipate. He validates her questions, and together, they problem-solve. They also set multiple reminders to help her remember the exposures they plan, and schedule follow-up sessions to provide support.

Neurohumility – Therapists working with socially anxious Autistic clients must remain aware of differences between their social experiences and those of the client, not assume that social exposures helpful for non-Autistic clients will work for Autistic ones, and curiously explore the individual Autistic client’s experiences and needs. Steve openly shares that while he has struggled with some social anxiety himself, he does not have the same experiences as Robin and he wants to understand her needs. He asks curious, nonjudgmental questions and validates her frequently. He also checks his interpretations and invites her to correct him.

Trauma informed – Socially anxious Autistic clients typically have social trauma histories as well as ongoing experiences of being bullied and rejected (122), which therapists need to understand to help plan therapeutic rather than re-traumatizing exposures. Steve curiously explores and validates Robin’s painful social history. They agree that while there is no guarantee that painful social experiences will not happen in the future, they will work together to help Robin build positive social experiences and connections and cope with difficulties.

## Discussion

While exposure therapies are highly effective for alleviating anxiety, they need to be tailored for the unique needs of Autistic clients to maximize benefit and avoid harm. The ASCENT model provides a guide that can help tailor exposure therapies to be more effective and affirming for Autistic clients (29, 30). In alignment with this model, it is essential for therapists using exposure therapies with Autistic clients to support client Autonomy in Affirming goal setting (A), support differences in Sensory, Stimming, Structure, Special interests (S), Communication (C), and Executive Functioning (E), practice with Neurohumility (N), and be Trauma informed (T). This article describes how the ASCENT model can guide tailoring exposure therapies to optimize these treatments for Autistic clients.

## Data Availability

The original contributions presented in the study are included in the article. Further inquiries can be directed to the corresponding author.
